# Changes in "natural antibiotic" metabolite composition during tetraploid wheat domestication

**DOI:** 10.1038/s41598-021-98764-5

**Published:** 2021-10-13

**Authors:** Yuval Ben-Abu, Mark Itsko

**Affiliations:** 1 Department of Physics and Project Unit, Sapir Academic College, 79165 Sderot, Hof Ashkelon Israel; 2 Clarendon Laboratory, Department of Physics, University of Oxford, Oxford, UK; 3 Present Address: WDS Inc., Contractor to Centers for Disease Control and Prevention, 1600 Clifton Road, Atlanta, GA 30033 USA

**Keywords:** Chemical biology, Ecology, Evolution

## Abstract

Gramineous plants protect their seeds from a variety of biotic stresses by producing toxic and deterrent secondary metabolites such as benzoxazinoids. It is unclear how the composition and abundance of these natural toxins has changed over the course of crop-plant domestication. To address this uncertainty, we characterized differences in metabolic levels of benzoxazinoids and their derivatives, between four lines of tetraploid wheat: wild emmer wheat (WEW), the direct progenitor of modern wheat; non-fragile domesticated emmer wheat (DEW), which was first domesticated about 11,000 years ago; the subsequently developed non-fragile and free-threshing durum landraces (LD); and modern durum (MD) varieties. Three-dimensional principal component analysis of mass spectrometry data of wheat metabolites showed with high resolution clear differences between metabolic profiles of WEW, DEW, and durum (LD + MD) and similarity in the metabolic profiles of the two durum lines (LD and MD) that is coherent with the phylogenetic relationship between the corresponding wheat lines. Moreover, our results indicated that some secondary metabolites involved in plant defense mechanisms became significantly more abundant during wheat domestication, while other defensive metabolites decreased or were lost. These metabolic changes reflect the beneficial or detrimental roles the corresponding metabolites might play during the domestication of three taxonomic subspecies of tetraploid wheat (*Triticum turgidum*).

## Introduction

Gramineous plants such as wheat (*Triticum aestivum*), rye (*Secale cereale*) and maize (*Zea mays*) produce an indole-derived class of toxic and deterrent secondary metabolites called benzoxazinoids to defend themselves against microbial pathogens, weeds, insects, or herbivores^1–11^. The major benzoxazinoids are 2,4-dihydroxy-1,4-benzoxazin-3-one (DIBOA) and its 7-methoxy derivative (DIMBOA), which are constitutively present in the vacuole as glucosides (DIBOA-Glc and DIMBOA-Glc)^6,12,13^.

Benzoxazinoids composition and abundance change throughout plant life cycle. For example, the abundance of their glucoside derivatives is at the greatest level soon after germination, decreasing thereafter to lower levels^14–16^. Relative benzoxazinoids abundance has also been shown to vary among plant crops (e.g., maize and wheat^17^), tissues (e.g., shoots and leaves^6^), growth habitats (e.g., arid and humid^6,18,19^), and even soil type (e.g., dry and wet^17,20–22^). In addition, dramatic differences in benzoxazinoids content have been identified in microscale evolutionary contexts^22^.

**Figure 1:**
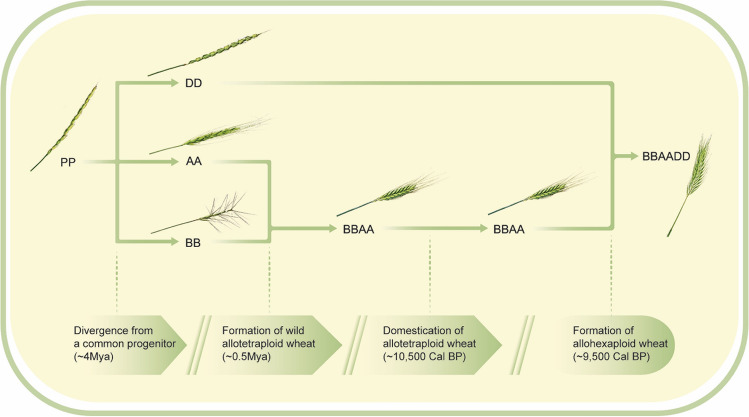
Evolutionary history of allotetraploid and allohexaploid wheat: Diploid wheats (2n = 2X = 14), from the *Tritcum-Aegilops* group have diverged ~ 4Mya from a diploid progenitor whose genome is indicated here as PP. Intergeneric hybridization between the diploid *T. urartu* (genome AA) as male and the donor of BB genome as female, (an unknown species similar to Ae. speltoides), followed by chromosome doubling, gave rise (~ 0.5Mya) to the wild allotetraploid wheat, *Triticum turgidum*,ssp. dicoccoides (genome BBAA, 2n = 4x = 28), the direct progenitor of durum and bread wheat. Domestication of allotetraploid wheat took place ~ 10,500 years ago and was followed by a second round of intergeneric hybridization chromosome doubling between domesticated allotetraploid wheat and the donor of the D genome, *Ae. Tauschii* (2n = 2X = 14, genome DD), giving rise, ~ 9000 years ago, to bread wheat, an allohexaploid (2n = 6X = 42, genome AABBDD).

Wheat domestication, the cornerstone of the agricultural revolution, took place about 11,000 years ago with the appearance of the first known form of domesticated emmer wheat (DEW, *Triticum turgidum* ssp*. dicoccum,* genome BBAA, 4x, 2n = 28) (Fig. 1). While DEW had been a prominent type of cultivated wheat for several millennia, today it exists only as a relatively minor crop, having been replaced, mostly during the Roman period, by durum wheat (*Triticum turgidum* ssp*. durum,* genome BBAA, 4x, 2n = 28), which is non-fragile and free-threshing^16,18,23^. Domesticated durum varieties can be classified into ancient landraces of durum (LD), which were selected by farmers, were locally adapted and grew under low input farming^19^, and modern durum (MD) varieties, dwarf and semidwarf lines, which were developed by plant breeders and grown on modern farms (Fig. 1). Hexaploid bread wheat (*Triticum aestivum* ssp *aestivum* genome BBAADD, 6x, 2n = 42) was formed ~ 9000 years ago through hybridization between a tetraploid (genome BBAA) and a diploid wheat, *Aegilops tauschii* (genome DD, 2x, 2n = 14), followed by whole-genome doubling (Fig. 1)^20,21^. Bread wheat became the most prominent wheat type grown nowadays, spreading from the fertile crescent to a wide range of environments, developing into the most extensively grown crop^21^.

Although the benzoxazinoids have been well investigated, the effects of domestication on these secondary metabolites and their derivatives remain unclear^14^). Indeed, previous studies of kernel metabolite composition during tetraploid wheat domestication have focused primarily on primary metabolites^24,25^, while studies of secondary metabolites have most frequently investigated the agronomic and environmental factors impacting these compounds^26–31^. Therefore, to our knowledge, no previous studies have characterized domestication-associated changes in the composition of secondary metabolites, particularly benzoxazinoids. To address the question as to how the profile of benzoxazinoids changed in the wheat during its domestication we compared levels of these metabolites in wild emmer (WEW), domesticated emmer (DEW) and durum in two types of plant tissue seed kernel and endosperm.

## Materials and methods

### Plant material

The study complies with local and national regulation. Nineteen accessions representing a total of four wheat lines corresponding to various stages of tetraploid wheat domestication were included in this study: five WEW accessions (*T. turgidum* ssp*. dicoccoides*), five DEW accessions, (*T. turgidum* ssp*. dicoccum*), five LD accessions (*T. turgidum* ssp*. durum*), and four MD accessions (*Triticum turgidum* ssp*. durum*; Table 1). Different wheat types were originally collected from traditional farmers from different countries as described in Table 1 and were stored at the seeds bank of Weizmann institute of science, Rehovot, Israel. All plants were grown in individual 3 L pots in a net house under identical conditions. Three replicates of each accession were grown, and each replicate was cultivated in a separate block. Nine wheat lines were grown in three parts of the greenhouse and analyzed according to the methods appeared below. Each part contained 4 season replicates.

**Table 1:** Tetraploid wheat lines used in this study.

Wild emmer wheat (WEW) *Triticum turgidum* ssp. *Dicoccoides*	Domestic emmer wheat (DEW) *Triticum turgidum* ssp. *Dicoccum*	Landraces durum (LD) *Triticum turgidum* ssp. *durum*	Modern durum (MD) *Triticum turgidum* ssp. *durum*
TTD20 (Israel)	Sub-group A: TTC1 (Italy)	TTR265 (Israel)	TTR16 (USA)
TTD32 (Turkey)	Sub-group A: TTC2 (India)	TTR2 (Israel)	TTR19 (Italy)
TTD43 (Syria)	Sub-group A: TTC8 (India)	TTR333 (Turkey)	TTR1 (Portugal)
TTD49 (Israel)	Sub-group B: TTC4 (Israel)	TTR5 (Morroco and Tunisia)	TTR25 (Israel)
TTD68 (Israel)	Sub-group B: TTC6 (Spain)	TTR6 (Israel)	

Country of origin for each line is shown in parentheses.

### Sample preparation

Each whole grain sample was separated into endosperm and embryo, and metabolite extraction was performed as described previously^23^. In brief, the embryo and endosperm tissues (500 mg each) were separately ground to a fine powder in liquid nitrogen, and 1.5 mL of 75% methanol with 0.1% formic acid was added. After sonication at room temperature for 15 min, each sample was centrifuged at 10,000 × *g* and filtered. The filtered samples were stored at − 20 °C prior to analysis. The metabolites in the extractable fractions were further purified using column chromatography (Amberlite XAD 8 HP) and eluted with ethanol. Both fractions were freeze-dried and stored at − 80 °C. The metabolite extraction was performed also from the isolated rye bran. The endosperm and embryo samples were extruded and hydrolyzed by xylanase treatment to release components from the bran matrix. A 10% water suspension of extruded endosperm and embryo were subjected to xylanase enzyme (Econase, AB Enzymes GmBH, Darmstadt, Germany), 5 U/g sample, 40 °C, for 21.5 h and cooled to 12–16 °C, followed by centrifugation and separation to extractable (water phase) and nonextractable (residue) fractions^23^. The metabolites in the extractable fraction were further purified by column chromatography (Amberlite XAD 8 HP) and eluted with ethanol. Both fractions were freeze-dried and stored at − 80 C. The metabolite extraction from the dried bran fractions was performed as follows: The extractable fraction was directly dissolved in 75% methanol with 0.1% formic acid at the ratio of 6 μL for 1 mg of dried sample. The residue was hydrolyzed by 1 N sodium hydroxide and incubated at 70 °C for 1 h, after which the pH was adjusted to 1–2 by adding 6 N hydrochloric acid. The sample was extracted three times with equal volumes of ethyl acetate and dried under vacuum. The sample was redissolved to 50% methanol and filtered prior to LC–MS analysis^32–35^.

### LC–MS metabolite analysis

Metabolite analysis was carried out using ultra-performance liquid chromatography coupled with a photodiode detector-quadrupole and tandem time-of-flight mass spectrometry (UPLC-PDA-qTOF-MS-Waters Premier qTOF, Milford, MA, USA). The system consisted of a Acquity UPLC (Waters) connected in-line to an Acquity PDA detector (Waters) and a Synapt HDMS detector (Waters). The HDMS system was operated in the standard qTOF mode, without using the ion mobility capabilities. Metabolite separation was performed using a UPLC BEH C18 column (100 × 2.1 mm i.d., 1.7 μm; Waters). The mobile phase consisted of 0.1% formic acid in acetonitrile/water (5:95, v/v) (phase A) and 0.1% formic acid in acetonitrile (phase B). The linear gradient program was as follows: 100–72% A over 22 min, 72–60% A over 0.5 min, 60–0% A over 0.5 min, holding at 100% B for a 1.5 min, then returning to initial conditions (100% A) over 0.5 min, and conditioning at 100% A. The flow rate was 0.3 mL/min, and the column temperature was kept at 35 °C. The UV spectra were recorded at 210–550 nm using the Acquity PDA detector (Waters), or the UV trace was measured at 240 nm using the Acquity UV detector (Waters). Eluting compounds were detected using the qTOF equipped with an electrospray ionization (ESI) source. Acquisition was performed in ESI-positive and ESI-negative modes. The following settings were applied during the LC–MS runs: capillary voltage, 3.0 kV; cone voltage, 30 eV; collision energy, 3 eV and 20 eV; and collision gas, argon. For the LCMS/MS analysis, collision energies of 20 and 35 eV were used. The m/z range was 50–1500 Da. The MS was calibrated using sodium formate, and leucine enkephalin was used as the lock mass. A standard mixture containing 40 μg/mL of each of the following compounds was used to monitor the quality of the chromatogram, to ensure the consistency of retention times across runs, and to aid in metabolite identification: L-tryptophan, L-phenylalanine, p-coumaric acid, caffeic acid, sinapic acid, benzoic acid, quercetin dehydrate, kaempferol, rutin, and trans-resveratrol (all purchased from Sigma); naringenin, chlorogenic acid hemihydrate, trans-cinnamic acid, and isorhamnetin (Fluka); ferulic acid (Aldrich); and tomatine (Apin chemicals). Mass Lynx v4.1 (Waters) was used to control all instruments and to calculate accurate masses^36–40^.

### LC–MS data analysis

The chromatograms obtained using UPLC PDA-qTOF-MS analysis were processed using Marker Lynx v4.1 (Waters) to extract and align mass signals. Metabolite identification was performed as described previously on data obtained using the ESI-negative mode^27^. In brief, accurate mass and molecular formula predictions for the putative molecules were screened against the Dictionary of Natural Products (Chapman and Hall/CRC) and the SciFinder Scholar databases (SciFinder Scholar 2007). The MS/MS fragmentation and UV-absorption of the metabolites were compared with those of candidate molecules found in databases and verified using previously published studies of similar compounds.

### Statistical analyses

Statistical analyses of the datasets were performed using Microsoft Excel 365 and MATLAB 8.0 with Statistics Toolbox 8.1. *P*-values (FDR) < 0.05 were considered significant. Figures were generated using MATLAB 8.0, GraphPad Prism 7.0, and Meta-Chart.

## Results

### Domestication-associated metabolomic alterations in wheat lines

**Figure 2:**
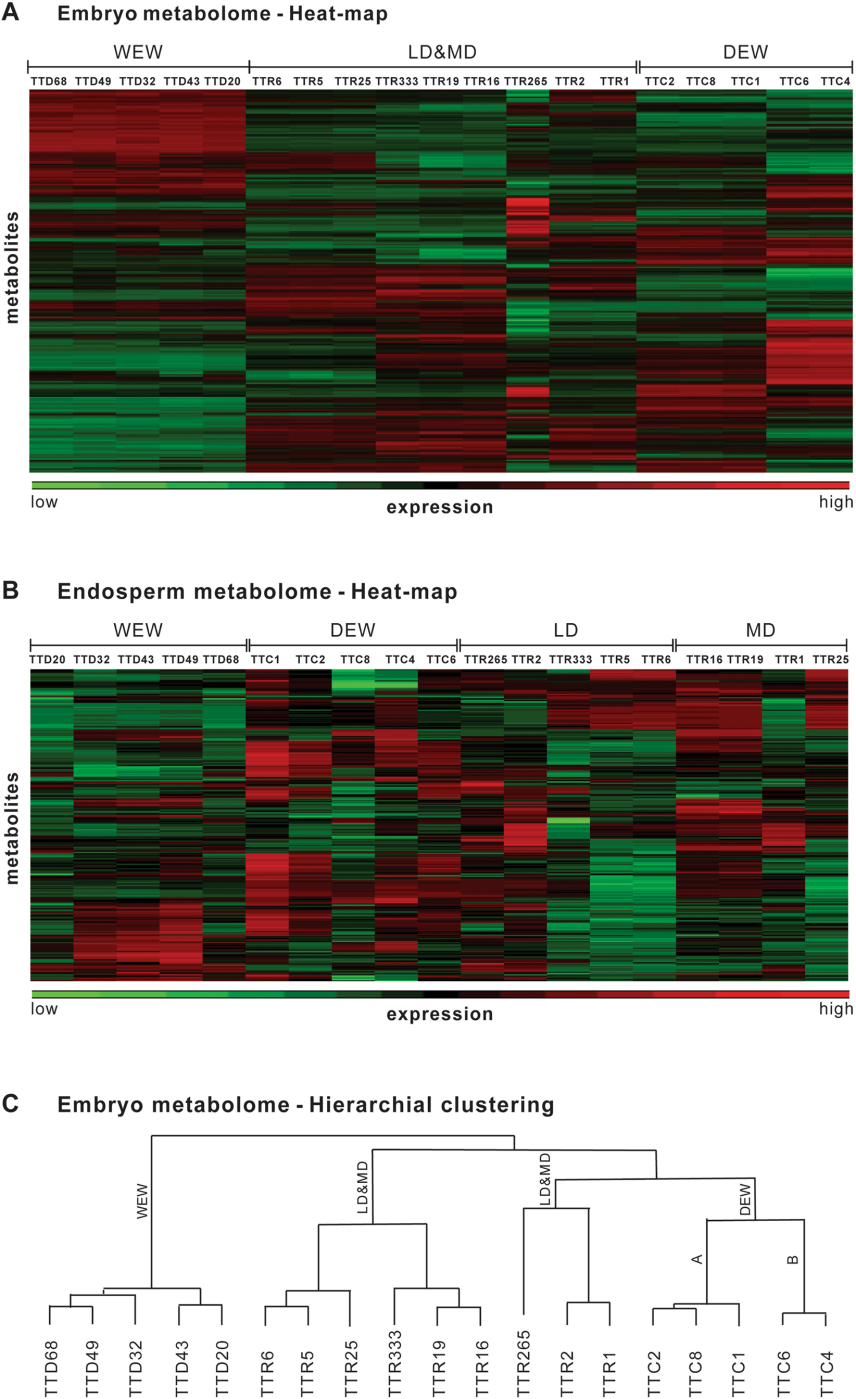
The embryo and endosperm metabolomes of 19 wheat accessions, representing three wild and domesticated tetraploid wheat subspecies at various stages of domestication: wild emmer wheat (WEW; *T. turgidum* ssp*. dicoccoides*), domesticated emmer wheat (DEW; *T. turgidum* ssp*. dicoccum*), durum landraces (LD; *T. turgidum* ssp*. durum*), and modern durum (MD; *Triticum turgidum* ssp*. durum*; see Table 1 for details of each accession). (**A**, **B**) Heat-maps of the (**A**) embryo and (**B**) endosperm metabolomes, generated based on the quantile-normalized average values of metabolites per accession. Red metabolites were upregulated, and green metabolites were downregulated. (**C**) Dendrogram showing relationships among the wheat embryo metabolomes based on the hierarchical clustering patterns of the heat-map shown in (A). The longer the Euclidean distance between two accessions, the greater the difference between the respective metabolomes.

We first compared metabolomes between the embryo and endosperm of 19 accessions, representing three wild and domesticated tetraploid wheat subspecies at various stages of domestication and from a variety of eco-geographical locations (Fig. 1 and Table 1). Across all accessions, LC–MS/MS analyses detected 4886 distinct metabolites in the embryo samples and 413 metabolites in the endosperm samples, showing that the embryo metabolome was much more complex than the endosperm metabolome. Next, we generated metabolite profiles for each accession by averaging metabolite values across replicas and normalizing the distributions. Tests of group effects identified 154 unique metabolites in the embryo samples and 9 unique metabolites in the endosperm samples (data not shown), again suggesting that the embryo metabolome was far more complex than the endosperm metabolome. Heatmaps of upregulated and downregulated metabolites in the embryo and the endosperm (Fig. 2A, B) revealed global patterns of metabolomic differences among the four wheat lines as well as consistent differences within lines. The dendrogram representing hierarchical clustering of the embryo metabolome heatmap showed distinct separations among the WEW, DEW, and durum lines (Fig. 2C). Interestingly, the durum accessions were divided into two subgroups, which did not correspond to LD and MD; the DEW accessions were also divided into two subgroups (Fig. 2C).

**Figure 3:**
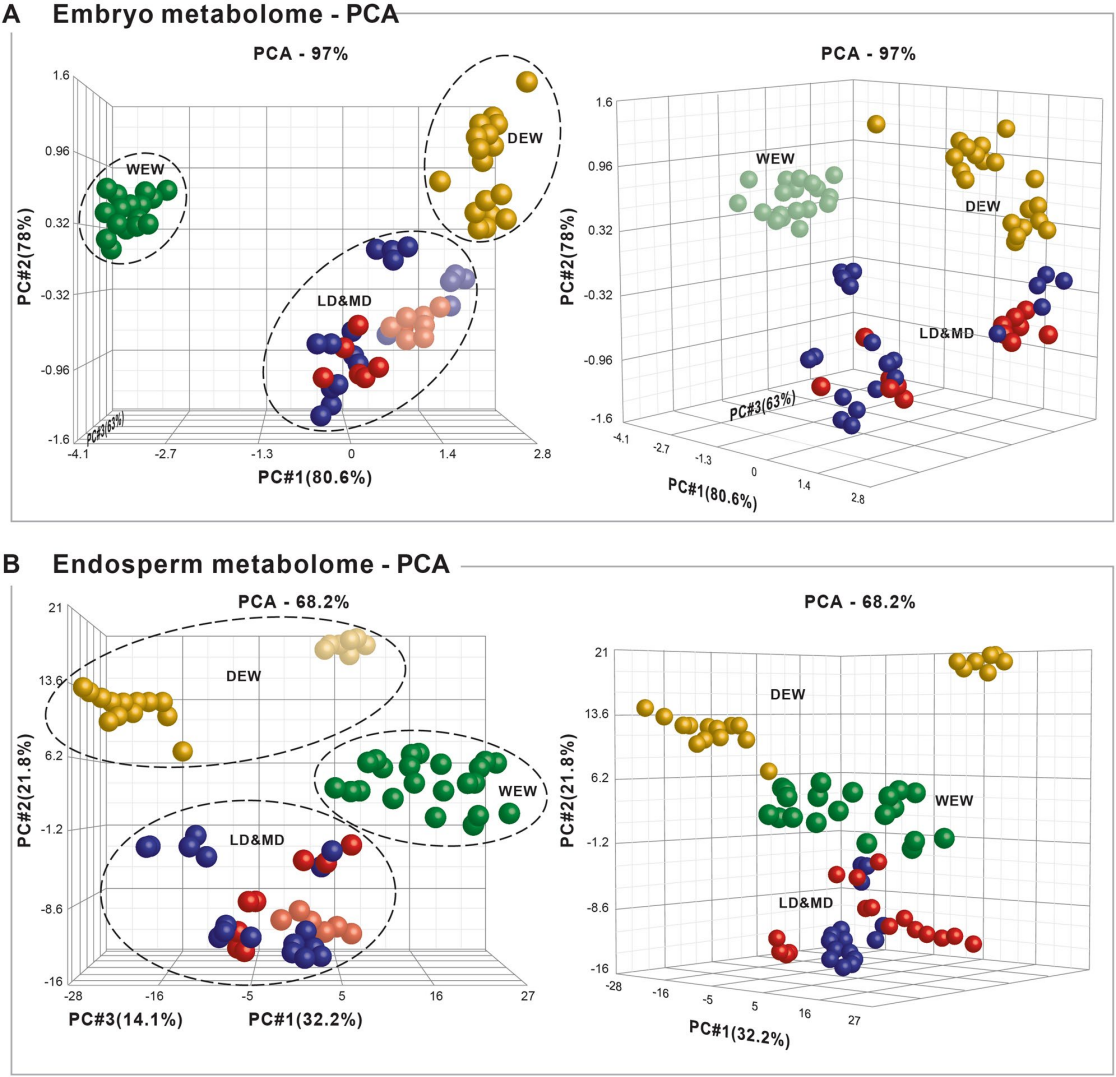
Three-dimensional models of principal component analyses (PCA) of the 19 wheat accessions, representing three wild and domesticated tetraploid wheat subspecies at various stages of domestication: wild emmer wheat (WEW; *T. turgidum* ssp*. dicoccoides*), domesticated emmer wheat (DEW; *T. turgidum* ssp*. dicoccum*), durum landraces (LD; *T. turgidum* ssp*. durum*), and modern durum (MD; *T. turgidum* ssp*. durum*; see Table 1 for details of each accession). Each sphere represents the quantile-normalized metabolome of one replicate accession; well-separated groups are indicated with dashed lines. (**A**) Two views of the PCA of the embryo metabolomes. (**B**) Two views of the PCA of the endosperm metabolomes.

Three-dimensional (3D) principal component analyses (PCAs), run without a wheat category-bias, also demonstrated a clear separation among the WEW, DEW, and durum accessions in both the embryo and the endosperm tissues (Fig. 2). Similar to the heatmaps, the 3D PCA models did not distinguish between the MD and LD accessions (Fig. 3). We again observed two distinct subgroups within the DEW accessions (Fig. 2). These DEW subgroups were designated A and B (Table 1). Thus, the heatmaps and the PCAs were consistent with our hypothesis that metabolite composition and expression underwent substantial changes during wheat domestication.

### Identification of benzoxazinoids metabolites associated with wheat domestication

To identify specific changes in the composition of benzoxazinoids, we searched the metabolites present in the wheat kernels against an annotated database^25^. The identification of the individual benzoxazinoid metabolites was based on analytical data and comparison to published LC–MS analyses on similar compounds. The most common chemical structures of benzoxazinoids include hydroxamic acids (2,4-dihydroxy1,4-benzoxazin-3-one, DIBOA; 2,4-dihydroxy-7-methoxy-1,4- benzoxazin-3-one, DIMBOA), lactams (2-hydroxy-1,4-benzoxazin-3-one, HBOA; 2-hydroxy-7-methoxy-1,4-benzoxazin-3-one, HMBOA), and benzoxazolinones (1,3-benzoxazol-2-one, BOA; 6-methoxy-1,3-benzoxazol-2-one, MBOA). We identified nine benzoxazinoids metabolites in the embryo samples and seven benzoxazinoids metabolites in the endosperm samples. We then characterized the metabolites that underwent significant changes during wheat domestication based on the averaged metabolomes of each group. Because there was no clear separation between LD and MD, we considered durum wheat a single group. We thus compared WEW to DEW, DEW to durum, and WEW to durum, with the WEW to durum comparison effectively summarizing the domestication process (Figs. 2 and 3).

**Figure 4:**
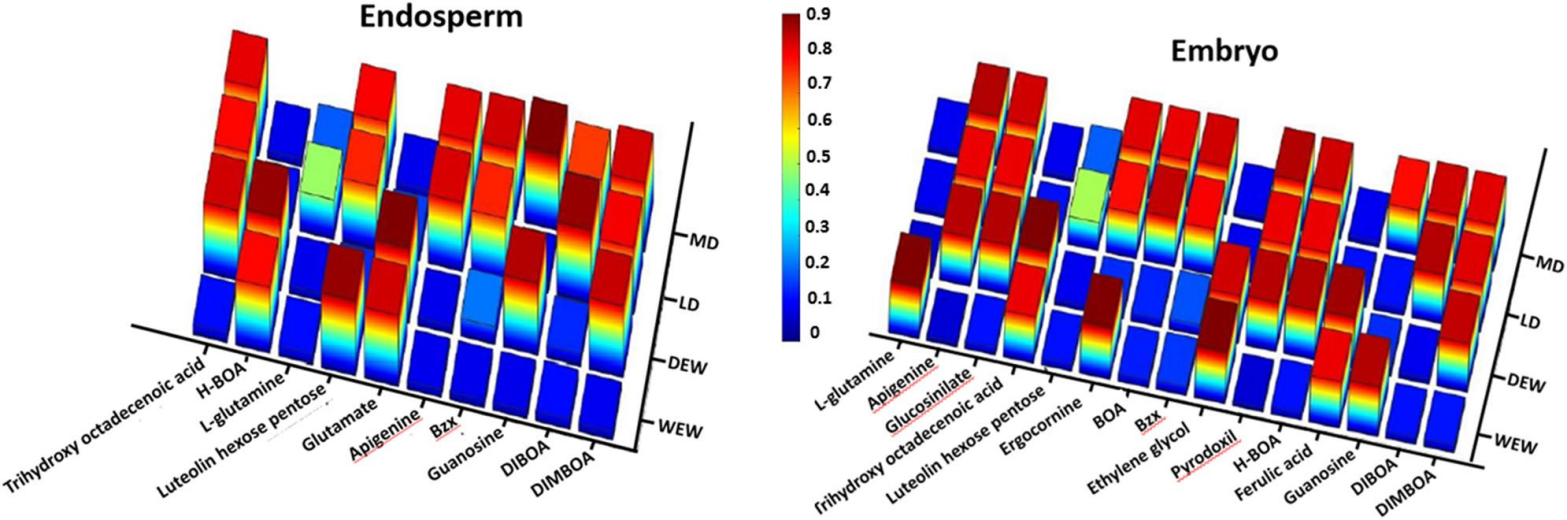
Changes in normalized benzoxazinoids metabolites-natural antibiotic metabolites profile during tetraploid wheat domestication in the (**A**) endosperm and (**B**) embryo.

Our examination revealed that generally higher concentrations of benzoxazinoids derivates such as HBOA, BOA, DIBOA and DIMBOA were found in different amounts in both the embryo and endosperm of DEW, LD and MD (Fig. 4). The endosperm of WEW showed lower amounts of these materials; in the endosperm of the other types such as LD and MD this HBOA totally disappears (Fig. 4). However, the HBOA amount in the embryo of WEW doesn’t appear and increases during the evolutionary stages in DEW, LD and MD. Furthermore, both in the endosperm and embryo, DIBOA doesn’t appear in WEW and DEW, and it increases in both MD and LD. Similar phenomena appear in DIMBOA, but they don’t appear in both the endosperm and embryo of WEW. Generally, the amounts of materials appear mainly in the embryo. In these kinds of materials, BOA appears only in the embryo of LD and MD.

## Discussion

Natural or artificial selection acts at the phenotypic rather than the genotypic level^41–46^. Therefore, the phylogenetic effect of selection can be measured based on some general observable property even in the absence of any knowledge about its genetic basis. In this study, we choose the whole wheat metabolome as a general phenotypic property to trace domestication-associated effects across the four lines of tetraploid wheat (Fig. 1). In addition, we specifically focused on domestication-associated changes in the composition and abundance of benzoxazinoids and its derivatives in the wheat lines. To trace both general and specific changes we performed an MS-coupled metabolomic analysis of the kernel embryo and endosperm to separate more than 4,000 metabolites including benzoxazinoids compounds and their derivatives. The seed tissues were chosen for the study because wheat domestication was focused on selection of useful seed traits which included seed size, its palatability, the loss of dormancy, seed dispersal mechanisms and its resistance against a variety of biotic stresses emerging in agro-ecosystems and differing from those in native habitats.

### General changes during domestication

Kernel embryo contained tenfold more metabolites compared to the endosperm. Notably, in the embryo, we detected greater levels of secondary metabolites associated with plant defense mechanisms and the response to biotic stress (Figs. 2 and 4), including phytoalexins, jasmonic acids, benzoxazinoids^4,47^, glucosinolates, and other alkaloids that act as bactericides^5,48^, fungicides and insecticides in various plants (Figs. 2, 3, and 4) . Clear divergences among three stages of domestication (WEW, DEW, and LD/MD) were observed in three dimensional PCAs, heatmaps, and evolutionary dendrograms of whole wheat metabolome (Fig. 3). No clear separation was observed between MD and LD, possibly due to the short time since the divergence of these groups (60–70 years; and/or a lack of selective pressure under modern farming practices^20,21,32–35,46^.

### Specific changes during domestication

We specifically investigated changes in the relative abundances of benzoxazinoids and its derivatives during wheat domestication. Our analyses revealed dramatic changes in benzoxazinoids composition over the course of wheat domestication, as evidenced by distinct separations between WEW, DEW, and durum wheat; benzoxazinoids profiles were similar between LD and MD. This remarkable separations between WEW, DEW, and durum highlighted the effects of domestication on benzoxazinoids components. Our results showed that the relative abundances of several antibiotic benzoxazinoids were altered in the wheat kernel endosperm and embryo during domestication. Indeed, we identified several metabolites neither previously described in the wheat metabolome, nor associated with domestication^25,47^.

Importantly, our comparative MS-coupled metabolomics analysis revealed that the relative abundances of benzoxazinoid metabolite derivates in the wheat kernel changed substantially during primary domestication (WEW to DEW) and secondary domestication (DEW to durum; Fig. 1). These shifts in composition and abundance may be due to climate change^40–42^, biotic stress, or other types of environmental alterations^6^. Levels of antibiotic substances were greater in different stages of wheat domestication, suggesting that some species during the wheat domestication survives and some disappear. Remarkably, levels of DIMBOA and its derivatives showed significant fluctuations during wheat domestication, may indicate to disappearance or survives of wheats type according to changes in the response to biotic stresses (e.g., fungi and other pathogens).

Specifically, benzoxazinoids were previously shown to participate in the stress response of WEW^6^. Actually, the appearance or disappearance of one of these benzoxazinoids, mainly in the embryo tissue that is important for the next generation, may indicate the plant’s resistance and survival^6^. Disease resistance is an adaptive trait that strongly affects crop productivity^32^. Interestingly, some defense-related metabolites (e.g., alkaloids) were downregulated in the MD lines as compared to the LD lines (Figs. 3 and 4). The use of industrial pesticides may have led to the downregulation of these endogenous pesticides^6,11,13,14,49,50^. The re-expression of such “lost” metabolites in modern lines might help to improve crop resistance, while minimizing dependence on harmful industrial pesticides^33^. Indeed, crop resilience might be most usefully improved by using genetic selection or engineering to increase the release of phytochemicals that confer resistance to biotic stress.

Both biotic (e.g., pathogen invasion) and abiotic stresses (e.g., nutrient deficiencies, extreme temperatures, and drought) may lead to oxidative stress in plants, against which antioxidants can provide protection^34^. Genes that induce antioxidant expression in wheat may also confer disease resistance^35,36^. In addition, high levels of antioxidants benefit the eventual consumers of wheat as a food source^37–39^. Our comparative metabolomics analysis revealed that antioxidants, including apigenin derivatives, flavonols, and glutathiones^34^, were greater in the kernels of the domesticated wheat lines as compared to the wild wheat (i.e., WEW; Fig. 4).

Overall, our results showed that benzoxazinoid production was substantially altered over the course of domestication (Fig. 4). Thus, our results may provide a reference for the identification of optimal breeding strains with pre-domestication benzoxazinoids profiles or with novel genes encoding for certain benzoxazinoids “lost” during domestication, the incorporation of which may improve wheat nutritional value or pest resistance.
